# Polyglycolic Acid Aerostatic Patch for Air Leak Management: Results from a Decade of Pulmonary Resections Using Propensity-Score Weighting

**DOI:** 10.1093/icvts/ivaf312

**Published:** 2025-12-27

**Authors:** Olivier Georges, Damien Basille, Julien Epailly, Florence de Dominicis, Paul-Emmanuel Esmard, Malek Ben Rahal, Alejandro Witte Pfister, Patrick Bagan, Pascal Berna, Osama Abou-Arab, Christophe Beyls

**Affiliations:** Thoracic Surgery Department, Amiens University Hospital, Amiens, F-80054, France; Department of Respiratory Disease and Critical Care Unit, Amiens University Hospital, Amiens, F-80054, France; Thoracic Surgery Department, Amiens University Hospital, Amiens, F-80054, France; UR UPJV 7518 SSPC (Simplification of Care of Complex Surgical Patients) Research Unit, University of Picardie Jules Verne, Amiens, 80000, France; Thoracic Surgery Department, Amiens University Hospital, Amiens, F-80054, France; Thoracic Surgery Department, Amiens University Hospital, Amiens, F-80054, France; Thoracic Surgery Department, Amiens University Hospital, Amiens, F-80054, France; Thoracic Surgery Department, Amiens University Hospital, Amiens, F-80054, France; Thoracic Surgery Department, Amiens University Hospital, Amiens, F-80054, France; Thoracic and Vascular Surgery Department, Victor Dupouy Hospital, Argentueil, 95018, France; Thoracic Surgery Department, Clinique Victor Pauchet, Amiens, France; Anesthesiology and Critical Care Department, Amiens University Hospital, Amiens, F-80054, France; UR UPJV 7518 SSPC (Simplification of Care of Complex Surgical Patients) Research Unit, University of Picardie Jules Verne, Amiens, 80000, France; Anesthesiology and Critical Care Department, Amiens University Hospital, Amiens, F-80054, France

**Keywords:** Neoveil, air leak, pulmonary resection, thoracic surgery, propensity score analysis

## Abstract

**Objectives:**

Prolonged air leaks are common after thoracic surgery and may be managed with synthetic aerostatic devices. This study assessed the impact of the Neoveil patch on air leak duration, hospital stay, and postoperative pneumonia.

**Methods:**

We conducted a retrospective monocentric study at Amiens University Hospital including adults undergoing lung resection between 2014 and 2024. Patients were divided into three groups: those receiving Neoveil, those not receiving it because of absence of indication, and those operated before its introduction in 2017. For analysis, the two latter groups were pooled as the Non-Neoveil arm. To address confounding, a propensity score was built from baseline covariates with standardized mean difference >5%, and inverse probability weighting was applied. The primary end-point was air leak duration, assessed with weighted linear regression. Secondary outcomes were hospital stay and postoperative pneumonia, analyzed with weighted linear and logistic regression.

**Results:**

Among 1216 patients, 313 (26%) received Neoveil. Compared with the control group, Neoveil use was associated with shorter air leak duration both before adjustment (−1.01 days; *P* = .0004) and after adjustment (−0.67 days; *P* = .0042). Hospital stay was also reduced (−1.88 days before adjustment; −1.09 days after adjustment; *P* = .0022). No significant difference was observed for postoperative pneumonia after adjustment (adjusted Odds Ratio 0.73, 95% Confidence Interval 0.48-1.11; *P* = .14).

**Conclusions:**

Neoveil use was associated with reduced air leak duration and shorter hospital stay following lung resection, without significant impact on pneumonia. These findings support its potential role in enhancing postoperative recovery and highlight the need for confirmation in prospective multicentre studies.

## Graphical abstract

**Figure:**
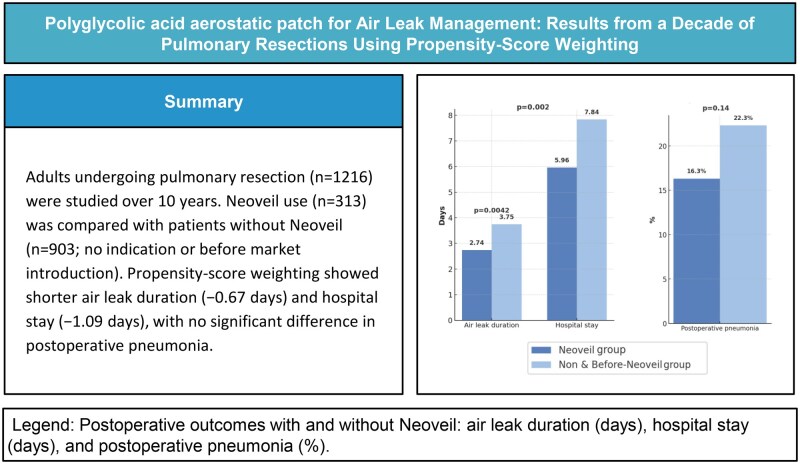

## INTRODUCTION

Regardless of the surgical approach, manipulation and dissection of the lung parenchyma in thoracic surgery lead to pleural breaches, resulting in air leaks. In the postoperative period, the persistence of these leaks requires maintaining a chest drain.

Numerous studies have demonstrated the negative impact of prolonged air leaks.^1–4^ Firstly, the presence of the drain reduces patient mobility and autonomy while causing parietal pain that requires the administration of analgesic drugs.^5^ Reduced mobility and pain themselves increase the risk of bronchial congestion, postoperative pneumonia, and thromboembolic events.^6^^,^^7^ Secondly, air leaks directly contribute to the risk of postoperative pleuropneumonia through bacterial translocation from the respiratory tract to the pleural cavity.^8^^,^^9^

In the era of ERAS, managing postoperative complications and reducing hospitalization duration is central to patient care. Thus, minimizing air leaks has become a key focus in thoracic surgery. In addition to optimizing surgical practices (careful manipulation, video-assisted surgery, fissureless dissection, etc), synthetic aerostatic devices are increasingly being utilized. This includes Neoveil (Gunze Ltd, Japan), an acid polyglycolic compress applied directly to the lung parenchyma to reinforce fragile areas and hypothetically reduce postoperative air leaks and drainage duration. Although Neoveil has been investigated in previous studies, evidence from large real-world cohorts and a historical reference population remains limited. Therefore, the primary end-point of this study was to assess the effectiveness of Neoveil patches in reducing the duration of air leaks.

The secondary end-points included assessing the impact of Neoveil on the duration of hospitalization and the occurrence of postoperative pneumonia (POP).

## METHODS

### Population study

This retrospective, single-center study included all adult patients hospitalized for suspected non-small cell lung cancer (NSCLC) undergoing major anatomical lung resection (bilobectomy, lobectomy, or segmentectomy) at Amiens-Picardie University Hospital. Procedures were performed via thoracotomy or video-assisted thoracic surgery (VATS). Exclusion criteria included non-anatomical resections (e.g., wedge resections), pneumonectomy, loss to follow-up, and cases with missing data for any of the main outcomes.

This study was designed to evaluate the effect of Neoveil while accounting for changes in surgical practices over time. We therefore compared three cohorts of patients who underwent pulmonary resection. The first was a historical cohort, referred to as the Before Neoveil group (January 2014-September 2017), representing a period when Neoveil was not yet available at our center, serving as a historical reference. After Neoveil became available (September 2017-April 2024), patients were divided into two contemporary cohorts: one that received Neoveil based on predefined intraoperative indications and one that did not receive Neoveil because none of these criteria were present. This three-group design was chosen to provide both a historical reference and a contemporary control group, allowing for a clearer evaluation of the effect of Neoveil.

### Indications for Neoveil application

In the contemporary population, Neoveil was applied based on predefined, strict indications that were consistently followed by all surgeons in the department. These included the intraoperative visualization of air leaks during ventilation testing (1), extensive fissure dissection (2), or the presence of macroscopically emphysematous lung parenchyma (3). Extensive fissure dissection was defined as the extensive separation of dense fissural adhesions or incomplete fissures leading to exposure of large parenchymal surfaces.

### Ethics

This retrospective study analyzes a prospective electronical database to investigate the clinical status after thoracic surgery. The registry and this study were reviewed and approved by the Amiens University Hospital Research Ethics Committee (reference DRCI-T38). In accordance with French law on clinical research for non-interventional studies, oral and written information are provided whenever possible to the patients and systematically to their families, specifying that they could oppose the use of their data.^10^

No biological samples or biobank were involved. Data were anonymized and used in accordance with French data protection laws and the WMA Declaration of Taipei. The report was in accordance with the guidelines on reports for propensity score analysis^11^ and STROBE statement for retrospective studies.^12^

### Description of the device

Neoveil is an absorbable reinforcement felt made of polyglycolic acid (PGA), marketed by Gunze Ltd, Japan. The sheet is applied as a single layer over the intended coverage area and secured on the lung surface with gentle pressure using damp pads for two min.

This device is CE-marked and widely used in several thoracic surgery departments across Europe and Japan for the prevention of postoperative air leaks. In our institution, Neoveil has been listed in the official hospital formulary and is available since September 2017. Its use in this study reflects routine clinical practice and is not part of a prospective interventional research protocol.

### Standard care in the study

All patients underwent elective thoracic surgery (lobectomy, bilobectomy, or segmentectomy) with systematic hilar and mediastinal lymph node dissection. Perioperative care followed a standardized ERAS protocol. Postoperatively, patients were transferred to the recovery room or, when warranted by comorbidities or surgical complexity, to a step-down intensive care unit for 24-48 h. Analgesia was multimodal, combining systemic drugs with regional techniques (paravertebral block or thoracic epidural for thoracotomy). Respiratory management included preoperative NIV in selected high-risk patients, early mobilization, physiotherapy, and incentive spirometry to optimize lung re-expansion and prevent atelectasis.

Chest drainage management followed departmental guidelines. Drains were connected either to a conventional suction system (PleurEvac, Teleflex Medical, USA) or to the Thopaz electronic system (Medela, Switzerland). Air leaks were monitored daily, and drain removal was performed once cessation of air leaks was confirmed and fluid output fell below 300 ml per 24 hours, with radiological confirmation to ensure lung re-expansion.

Patients underwent twice-daily assessments (auscultation, temperature) and routine chest X-rays until drain removal and discharge; a standardized pathway applied across groups ensured comparability and minimized perioperative management variability.

### Outcomes

The primary outcome was the duration of postoperative air leaks, defined as the number of days until their cessation. Air leaks were measured in days, and were monitored daily by the ward nurses. The absence of air leaks was confirmed either by the absence of bubbling in traditional drainage systems or by the absence of flow on the Thopaz Medela electronic drainage system.

Secondary outcomes included the length of hospital stay, measured in days from surgery to discharge, and the incidence of postoperative pneumonia (POP). POP was defined based on a standardized composite criterion requiring a pulmonary infiltrate on chest X-ray accompanied by at least two of the following: fever (1), persistent or new postoperative inflammatory syndrome (2), or clinical symptoms (3) such as focal auscultatory findings, purulent sputum, or dyspnoea. Additionally, 30- and 90-day postoperative mortality were assessed based on clinical records and follow-up data.

### Data collection

Baseline demographic, clinical, and functional characteristics were extracted from institutional record.

Intraoperative variables included surgical approach, procedure type, and conversion to thoracotomy.

Postoperative outcomes comprised air-leak duration, length of stay, postoperative pneumonia, bronchopleural fistula, reintubation, and 30- and 90-day mortality.

All data were entered prospectively into a secure, standardized institutional registry maintained by the thoracic surgery department. This registry is updated in real-time by dedicated clinical research personnel and surgical staff, with systematic verification from the medical records and operative reports.

Postoperative status at 30 and 90 days was assessed through standardized follow-up combining in-hospital records, reports from referring physicians, and, when needed, structured telephone interviews. Mortality data were verified through multiple sources and confirmed via the national death registry (INSEE).

### Missing data and propensity score

Among the 1326 patients initially screened, 80 were excluded due to missing data on the main outcomes (air-leak duration, hospital stay, or postoperative pneumonia). These cases were not imputed. In the remaining dataset (*n* = 1216), several baseline variables showed limited missingness, mainly albumin (6.8%), pre-operative FEV_1_ (4.5%), body mass index (3.9%), smoking status (2.5%), and anaesthesia duration (3.2%). Missing data were assumed to be missing at random (MAR) and were imputed using predictive mean matching according to Rubin’s multiple imputation framework.^13^

### Statistical analysis

Data were expressed as mean ± standard deviation (SD) or median [interquartile range] for continuous variables and as numbers (percentages) for categorical variables. To account for confounding, a propensity score (PS) was constructed to estimate the probability of receiving Neoveil for each patient. The PS model included all clinically relevant variables and those showing initial imbalance between the Neoveil and no-Neoveil groups, defined as a standardized mean difference (SMD) > 5%. The covariates included were: gender, ENT history, thoracic radiotherapy, congestive heart failure, cardiac arrhythmia, COPD, FEV1, history of smoking, smoking cessation, BMI, left-sided procedure, VATS approach, and type of resection.

A logistic regression model was used to estimate the PS, and stabilized inverse probability of treatment weighting (IPTW) was applied to create a pseudo-population with balanced baseline covariates. Post-weighting, covariate balance was reassessed, with an SMD < 10% considered acceptable.^14^ The outcomes were then analysed using weighted logistic regression for binary variables and weighted linear regression for continuous variables.

Statistical analyses were performed using Python (version 3.9) with pandas, statsmodels, and matplotlib packages. A two-sided *P*-value of less than .05 was considered statistically significant. Reported p values were unadjusted for multiplicity.

Reporting followed the STROBE recommendations for observational studies.

## RESULTS

### Population study (Figure 1)

From January 10, 2014, to April 30, 2024, a total of 1326 patients underwent anatomical lung resection surgery for non-small cell lung cancer (NSCLC). Among them, 120 patients were excluded due to missing data on one of the variables of interest. Finally, 1216 patients were included in the analysis, with 313 (25.7%) receiving the Neoveil patch.

### Baseline and intraoperative characteristics (Table 1)

**Table 1.** Descriptive Analysis of the Patients’ Pre-Operative & per Operative Characteristics

Variable	Neoveil group	Non-Neoveil group	Before Neoveil group	*P*-value
Age; years	64.11 ± 8.93	64.45 ± 9.55	63.65 ± 9.76	.632
BMI; kg/m^2^	26.49 ± 4.60	25.94 ± 4.41	26.26 ± 4.99	.294
Male gender; *n* (%)	218 (69.6)	272 (74.7)	417 (77.4)	.044
ENT cnacer; *n* (%)	44 (14.1)	30 (8.2)	35 (6.5)	**<.001**
Thoracic radiotherapy; *n* (%)	22 (7.0)	18 (4.9)	28 (5.2)	.433
Induction chemotherapy; *n* (%)	39 (12.5)	38 (10.4)	71 (13.2)	.460
Diabetes; *n* (%)	52 (16.6)	67 (18.4)	82 (15.2)	.447
Hypertension; *n* (%)	135 (43.1)	172 (47.3)	227 (42.1)	.296
Congestive heart failure; *n* (%)	7 (2.2)	6 (1.6)	22 (4.1)	.073
Coronary artery disease; *n* (%)	41 (13.1)	62 (17.0)	62 (11.5)	.056
Cardiac arrhythmia; *n* (%)	17 (5.4)	38 (10.4)	48 (8.9)	.058
COPD; *n* (%)	98 (31.3)	106 (29.1)	212 (39.3)	**.002**
Renal failure; *n* (%)	11 (3.5)	17 (4.7)	20 (3.7)	.692
FEV1	83.98 ± 20.05	82.03 ± 17.40	81.55 ± 19.07	.204
History of smoking; *n* (%)	309 (98.7)	362 (99.5)	535 (99.3)	.556
Weaned smoking; *n* (%)	240 (76.7)	298 (81.9)	436 (80.9)	.199
Left-sided procedure; *n* (%)	118 (37.7)	161 (44.2)	237 (44.0)	.144
VATS; *n* (%)	263 (84.0)	238 (65.4)	324 (60.1)	**<.001**
Conversion; *n* (%)	37 (11.8)	55 (15.1)	55 (10.2)	.084
Intervention type				**<.001**
Lobectomy; *n* (%)	262 (83.7)	257 (70.6)	415 (77.0)	
Bilobectomies; *n* (%)	17 (5.4)	70 (19.2)	70 (13.0)	
Segmentectomy; *n* (%)	34 (10.9)	37 (10.2)	54 (10.0)	

Bold values indicate *p* ≤ 0.05.

The majority of the patients were male, with a slightly higher proportion in the Non-Neoveil and Before-Neoveil groups (74.7% and 77.4% vs 69.6%, *P* = .045). The median age of patients was similar across the three groups, with no significant difference observed.

Regarding medical history, the prevalence of ENT diseases was significantly higher in the Neoveil group compared to the Non-Neoveil and Before-Neoveil groups (14.1% vs 8.2% and 6.5%, *P* = .001). However, no significant differences were observed for other variables.

Similarly, the type of resection was balanced between groups. The Non-Neoveil group showed a higher rate of conversion from VATS to thoracotomy and had a slightly lower preoperative FEV1.

### Postoperative outcomes (Table 2)

**Table 2.** Postoperative Outcomes

Variables	Neoveil group	Non-Neoveil group	Before Neoveil group	*P*-value
Air leak duration; days	2.74 ± 3.67	3.40 ± 3.99	3.99 ± 4.88	**<.001**
Hospital LOS; days	5.96 ± 5.49	7.34 ± 5.43	8.18 ± 7.44	**<.001**
Post-operative pneumonia; *n* (%)	51 (16.3)	88 (24.3)	110 (20.4%)	.038
Endo tracheal reintubation; *n* (%)	6 (1.9)	10 (2.8)	38 (7.1)	**<.001**
Post-operative fistula; *n* (%)	5 (1.6)	5 (1.4)	10 (1.9)	.858
30-day mortality; *n* (%)	1 (0.3)	2 (0.6)	6 (1.1)	.383
90-day mortality; *n* (%)	3 (1.0)	2 (0.6)	18 (3.3)	**.004**

Bold values indicate *p* ≤ 0.05.

Patients with Neoveil had a significantly shorter drainage duration compared to the Non-Neoveil and Before-Neoveil groups, with a mean duration of 2.74 ± 3.67 days in the Neoveil group versus 3.40 ± 3.99 days and 3.99 ± 4.88 days in the Non-Neoveil and Before-Neoveil groups, respectively (*P* < .001). Similarly, the hospital length of stay was significantly shorter in the Neoveil group (5.96 ± 5.49 days) compared to the Non-Neoveil (7.34 ± 5.43 days) and Before-Neoveil groups (8.18 ± 7.44 days) (*P* < .001).

The rate of pneumonia was significantly lower in the Neoveil group (16.3%) compared to the Non-Neoveil group (24.3%) (*P* = .038). Reintubation rates were also significantly lower in the Neoveil group (1.9%) compared to both the Non-Neoveil (2.8%) and Before-Neoveil (7.1%) groups (*P* = .0004).

No statistically significant differences were observed between groups regarding postoperative fistula rates (*P* = .8583) or 30-day mortality (*P* = .3839). However, the 90-day mortality rate was significantly higher in the Before-Neoveil group (3.3%) compared to the Neoveil (1.0%) and Non-Neoveil (0.6%) groups (*P* = .0040).

### Association between Neoveil use and end-points before and after IPW analysis (Table 3—Table S1)

**Table 3.** Association between Neoveil Use and Clinical Endpoints

Endpoint	Neoveil group	Non & Before Neoveil group	Before IPW (effect estimate, *P*-value)	After IPW (effect estimate, *P*-value)
Air leak duration (days)	2.74 ± 3.67	3.75 ± 4.3	−1.01 days (*P* = .0004)	−0.67 days (*P* = .0042)
Hospital stay (days)	5.96 ± 5.49	7.84 ± 6.4	−1.88 days (*P* = 8.78 × 10⁻⁶)	−1.09 days (*P* = .0022)
Postoperative pneumonia	51 (16.3%)	88-110 (22.3%)	OR = 0.60 [0.40-0.90], *P* = .015	OR = 0.73 [0.48-1.11], *P* = .14 (NS)

Thirteen variables were identified as potential confounders, with a standardized mean difference (SMD) greater than 5% before propensity score weighting. After IPW, these confounders were adequately balanced, as illustrated in **Figure 2** (Love Plot). Detailed SMD values before and after weighting are provided in **Table S1**.

**Figure 1.**
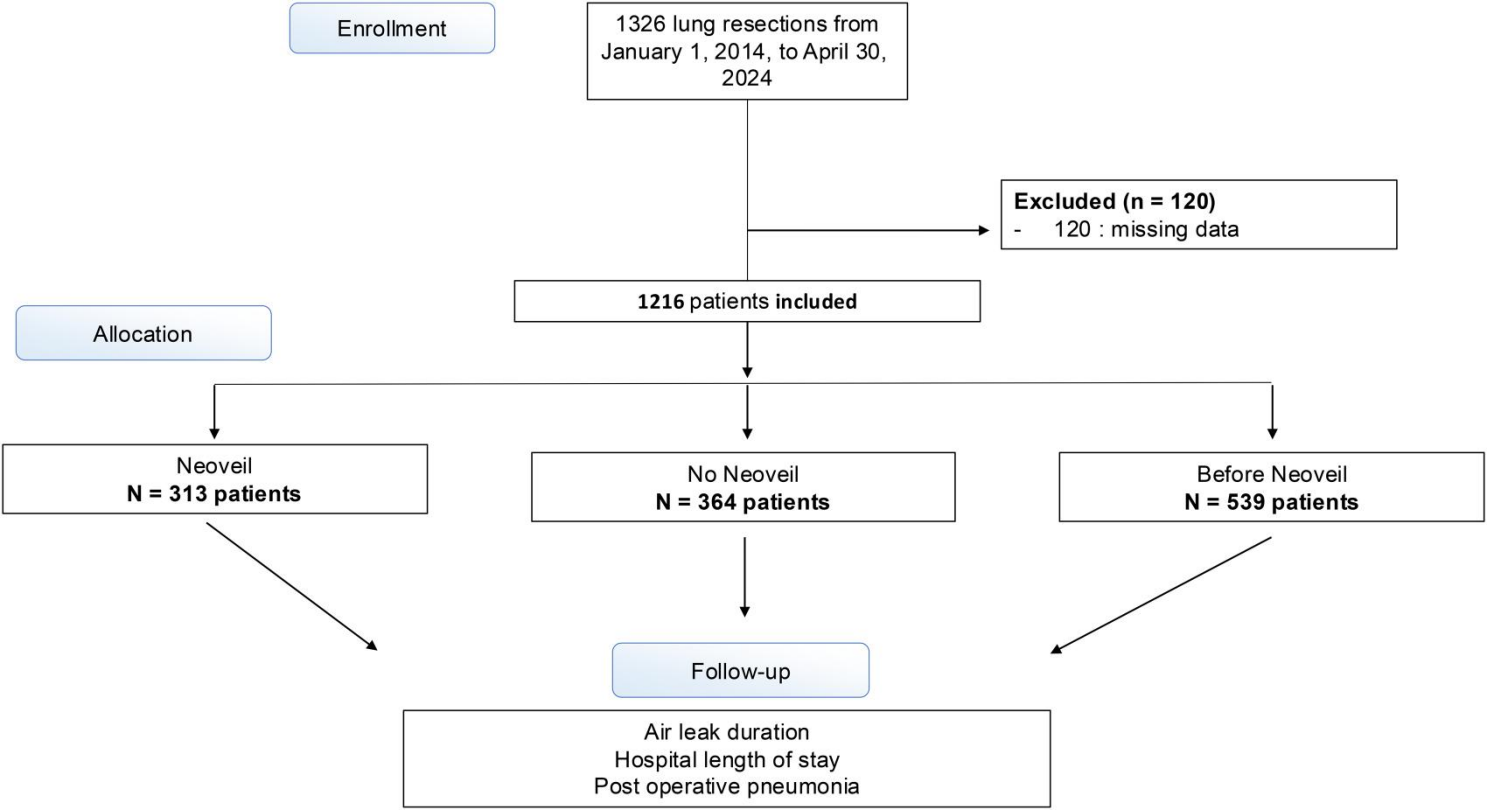
Study Flow Chart of Patient Inclusion and Group Allocation

**Figure 2.**
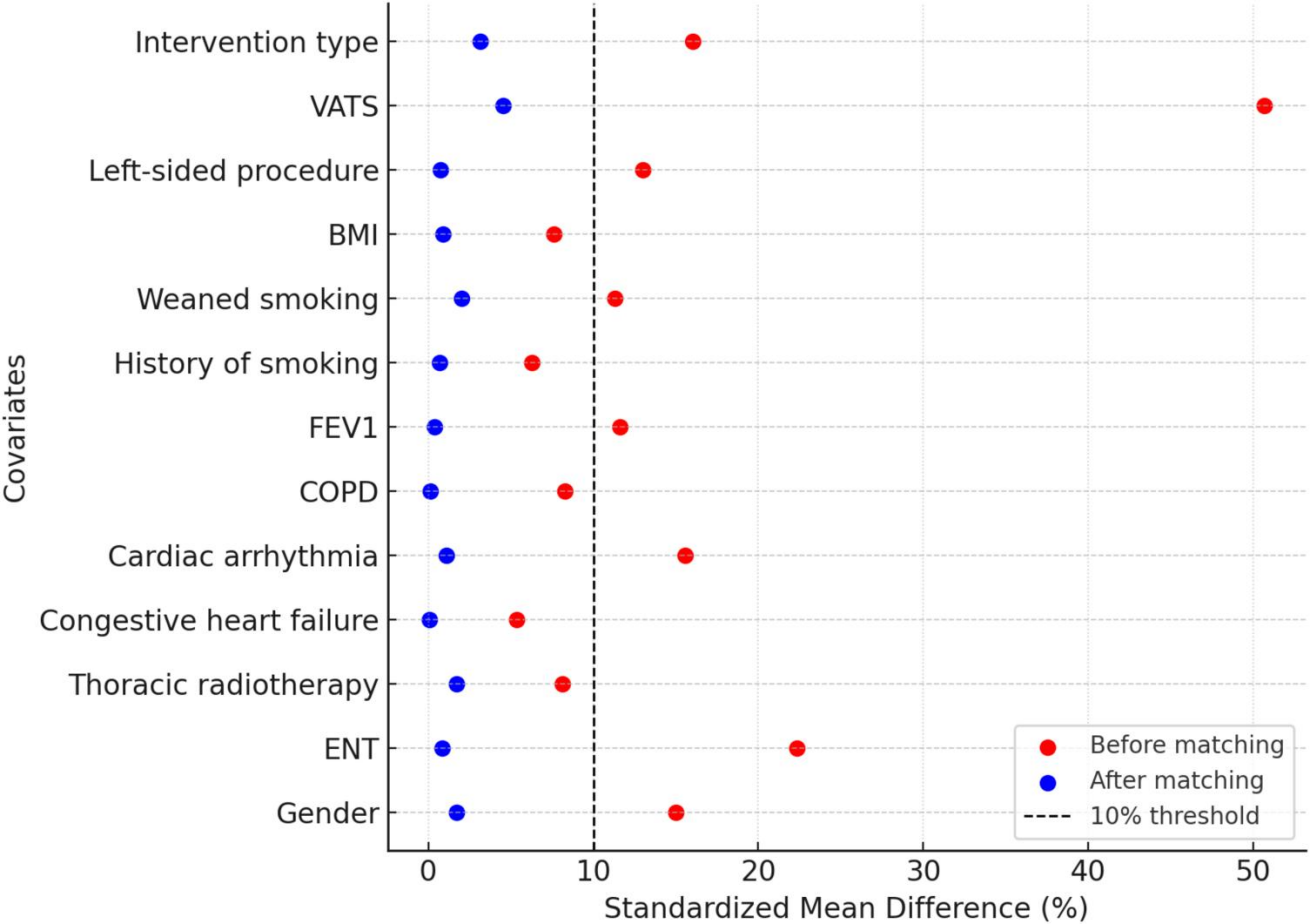
Love Plot Showing Standardized Mean Differences before and after IPW

For the analysis of clinical end-points with IPW weighting, data from the Non-Neoveil and Before-Neoveil groups were pooled to form a single control arm, which was then compared with the Neoveil group (**Table 3**).

Before IPW, the Non-Neoveil group had a mean air leak duration of 3.75 days. Linear regression revealed that Neoveil use was associated with a significant reduction of 1.01 days (*P* = .0004). After IPW, this association remained significant, reducing the duration by 0.67 days (*P* = .0042).

Similarly, before IPW, the mean hospital stay in the Non-Neoveil group was 7.84 days. Linear regression showed that Neoveil use was associated with a reduction of 1.88 days (*P* = 8.78 × 10^−6^). After IPW, this association was confirmed, reducing the length of stay by 1.09 days (*P* = .0022).

Regarding postoperative pneumonia, logistic regression before IPW indicated that Neoveil use was associated with a significantly lower risk (OR = 0.60 [0.40; 0.90], *P* = .015). However, after IPW, this association was no longer significant (OR = 0.73 [0.48; 1.11], *P* = .14).

Sensitivity analyses restricted to the contemporary cohort (2017-2024) yielded similar directional trends (**Table S2**).

## DISCUSSION

### Key findings

In this population-based observational propensity score-weighted study among thoracic surgery patients, the use of the Neoveil patch was associated with a significantly shorter duration of air leaks and reduced hospital stay.

### Clinical significance

Although the absolute reductions after IPW (−0.67 day for air leak and −1.09 days for length of stay) may appear modest, they remain clinically meaningful within ERAS frameworks. Even a one-day decrease in drainage duration or hospitalization can facilitate earlier mobilization, improve patient comfort, and reduce nosocomial exposure. From a healthcare perspective, shorter postoperative stays also contribute to better bed turnover and lower costs, which are particularly relevant in high-volume thoracic surgery units.

Regarding postoperative pneumonia rates, the crude reduction initially observed with Neoveil was likely confounded by baseline differences; after IPW adjustment, no independent association was found.

### Strenghts & limitations

The statistical approach, using propensity score weighting with inverse probability weighting (IPW), adjusts for confounders and limits bias. Effective balancing of baseline characteristics, demonstrated by the Love Plot and standardized mean difference (SMD) analysis, supports comparability between groups and strengthens the reliability of associations.

Another strength is the inclusion of a historical cohort treated before 2017, when Neoveil was unavailable. This cohort serves as a reference by capturing outcomes unrelated to clinical contraindications. Its integration into the Non-Neoveil group broadens the comparator arm and reduces bias linked to selective use.

The study also benefits from a large, real-world cohort of 1216 patients over nearly a decade, reflecting heterogeneity in surgical techniques, perioperative management, and patient characteristics. This diversity enhances generalizability and provides insights into Neoveil use across varied scenarios.

Nevertheless, limitations must be acknowledged. This observational design cannot establish causality, and unmeasured variables may influence results. Although the IPW approach balanced all measured confounders, residual confounding by indication cannot be excluded, as Neoveil was systematically applied in patients with the most fragile parenchyma or visible air leaks. Therefore, unmeasured intraoperative factors may have contributed to the observed association.

These results demonstrate a significant association rather than a proven causal effect, and a randomized controlled trial is warranted to confirm this hypothesis.

Including the historical ‘Before-Neoveil’ cohort offered a reference population free from indication bias but may have introduced temporal confounding, as global improvements in perioperative care could have contributed to better outcomes independently of the device.

The retrospective single-center design may also lead to inaccuracies.

### Literature data

Other aerostatic agents are available on the market; however, no consensus has emerged from various recent studies. The most extensive study, published in 2018 by Mc Guire,^15^ was a meta-analysis including 2537 patients and revealed a one-day decrease in length of stay, duration of air leak, and duration of chest tubes with the addition of polymeric surgical sealant to standard care. However, none of the studies included in this pooled analysis used Neoveil.

Since then, a few studies have specifically investigated Neoveil, yielding contrasting effectiveness figures.^16^^,^^17^ However, those reports used smaller cohorts; or less robust statistical methods, such as logistic regression or matching, to adjust for confounding factors relative to a propensity-matched analysis.

*Generalisability* Our findings provide valuable insights into the potential impact of Neoveil in thoracic surgery. However, the single-center design and the specific clinical context may limit their applicability to other settings. While the results suggest promising benefits, further multicentre studies are needed to confirm these observations and assess their relevance across diverse surgical practices.

## CONCLUSION

Our findings suggest that the use of Neoveil is associated with a reduction in air leak duration and hospital stay following lung resection surgery. These results indicate that Neoveil may enhance postoperative recovery, but no significant impact was observed on the rate of postoperative pneumonia. Further prospective and multicentre studies are needed to confirm these findings and better define the clinical contexts in which Neoveil provides the greatest benefit.

## Supplementary Material

ivaf312_Supplementary_Data

## Data Availability

The data underlying this article will be shared on reasonable request to the corresponding author.
