# Supplementary material for: Polyglycolic Acid Aerostatic Patch for Air Leak Management: Results from a Decade of Pulmonary Resections Using Propensity-Score Weighting
Source: Interdiscip Cardiovasc Thorac Surg. 2025 Dec 27;41(1):ivaf312. doi: 10.1093/icvts/ivaf312 (PMC12823547; doi:10.1093/icvts/ivaf312)
Supplement: ivaf312_Supplementary_Data [file ivaf312_supplementary_data.docx]

**Supplementary Table 1. Summary of Standardized Mean Differences (SMD) Before and After Inverse Probability Weighting (IPW)**

| **Covariable** | **SMD Before PS (%)** | **SMD After PS (%)** |
| --- | --- | --- |
| Gender | 15.005 | 1.709 |
| ENT | 22.367 | 0.852 |
| Thoracic radiotherapy | 8.104 | 1.693 |
| Congestive heart failure | 5.359 | 0.059 |
| Cardiac arrhythmia | 15.591 | 1.078 |
| COPD | 8.288 | 0.121 |
| FEV1 | 11.602 | 0.365 |
| History of smoking | 6.2510 | 0.695 |
| Weaned smoking | 11.313 | 2.005 |
| BMI | 7.602 | 0.902 |
| Left-sided procedure | 12.981 | 0.723 |
| VATS | 50.661 | 4.508 |
| Intervention type | 16.0139 | 3.157 |

**Supplementary Table 2. Sensitivity analysis restricted to the contemporary cohort (2017–2024): weighted comparison between Neoveil™ and non-Neoveil groups after inverse probability weighting (IPW).**

| **Endpoint** | **Neoveil™ group** | **Non‑Neoveil group** | **Effect estimate** | **p-value** |
| --- | --- | --- | --- | --- |
| Air leak duration (days) | 2.85 ± 3.56 | 3.33 ± 4.00 | -0.48 days | 0.055 |
| Hospital stay (days) | 6.17 ± 5.47 | 6.87 ± 5.10 | -0.70 days | 0.086 |
| Postoperative pneumonia | 17.0% | 22.1% | OR 0.72 | 0.100 |

**Supplementary material : STROBE Statement Checklist for Cohort Studies**

| **Item No.** | **Recommendation** | **Reported in Manuscript (section/line)** |
| --- | --- | --- |
| **Title and abstract** |  |  |
| 1a | Indicate the study design with a commonly used term in the title or the abstract. | Title & Abstract: “retrospective cohort study” |
| 1b | Provide in the abstract an informative and balanced summary of what was done and what was found. | Abstract |
| **Introduction** |  |  |
| 2 | Explain the scientific background and rationale for the investigation being reported. | Introduction, first paragraph |
| 3 | State specific objectives, including any prespecified hypotheses. | Introduction, last paragraph |
| **Methods** |  |  |
| 4 | Present key elements of study design early in the paper. | Methods, first paragraph |
| 5 | Describe the setting, locations, and relevant dates, including periods of recruitment and follow-up. | Methods: “Amiens University Hospital, 2014–2024” |
| 6a | Give the eligibility criteria and the sources and methods of selection of participants. | Methods – Study population |
| 6b | For matched studies, give matching criteria and number of exposed and unexposed. | Not applicable (weighted analysis) |
| 7 | Clearly define all outcomes, exposures, predictors, confounders, and effect modifiers. | Methods – Outcomes & Covariates |
| 8 | For each variable of interest, give sources of data and details of measurement. | Methods – Data collection |
| 9 | Describe any efforts to address potential sources of bias. | Methods – Propensity score, IPW adjustment |
| 10 | Explain how the study size was arrived at. | Methods – Entire eligible population over 10 years |
| 11 | Explain how quantitative variables were handled in the analyses. | Methods – Statistical analysis |
| 12a | Describe all statistical methods, including those used to control for confounding. | Methods – Statistical analysis (IPW) |
| 12b | Describe any methods used to examine subgroups and interactions. | Not applicable |
| 12c | Explain how missing data were addressed. | Methods – Missing data paragraph |
| 12d | If applicable, explain how loss to follow-up was addressed. | Not applicable (retrospective) |
| 12e | Describe any sensitivity analyses. | Results – Sensitivity analysis paragraph |
| **Results** |  |  |
| 13a | Report numbers of individuals at each stage of study (e.g., eligible, included, analyzed). | Flowchart (Figure 1) |
| 13b | Give reasons for non-participation at each stage. | Methods & Flowchart |
| 14a | Give characteristics of study participants (e.g., demographic, clinical, social) and information on exposures and potential confounders. | Table 1 |
| 14b | Indicate number of participants with missing data for each variable of interest. | Table 1, Methods – Missing data |
| 15 | Report numbers of outcome events or summary measures over time. | Results section |
| 16a | Give unadjusted and confounder-adjusted estimates and precision (e.g., 95% CI). | Results – Main outcomes |
| 16b | Report category boundaries when continuous variables were categorized. | Methods – Covariate definitions |
| 17 | Report other analyses done, e.g., sensitivity analyses. | Results – Sensitivity analysis |
| **Discussion** |  |  |
| 18 | Summarize key results with reference to study objectives. | Discussion – Key findings |
| 19 | Discuss limitations of the study, taking into account potential sources of bias or imprecision. | Discussion – Strengths & Limitations |
| 20 | Give a cautious overall interpretation of results considering objectives, limitations, and other relevant evidence. | Discussion – Strengths & Limitations & Clinical significance |
| 21 | Discuss the generalizability (external validity) of the study results. | Discussion – Generalisability |
| **Other information** |  |  |
| 22 | Give the source of funding and the role of the funders for the present study. | End of manuscript – Funding & Conflicts of interest |
